# Adverse Events Reported From Hyaluronic Acid Dermal Filler Injections to the Facial Region: A Systematic Review and Meta-Analysis

**DOI:** 10.7759/cureus.38286

**Published:** 2023-04-29

**Authors:** Jessica Colon, Sophia Mirkin, Patrick Hardigan, Matthew J Elias, Robin J Jacobs

**Affiliations:** 1 Dr. Kiran C. Patel College of Osteopathic Medicine, Nova Southeastern University, Fort Lauderdale, USA; 2 Health Professions Division, Nova Southeastern University, Dr. Kiran C. Patel College of Allopathic Medicine, Fort Lauderdale, Fl, USA; 3 Dermatology, Elias Dermatology, LLC, Fort Lauderdale, USA

**Keywords:** cosmetic procedures, facial region, adverse events, dermal fillers, hyaluronic acid

## Abstract

Dermal filler injections are one of the most popular cosmetic procedures in the United States. Of the many options available, hyaluronic acid (HA) dermal fillers like Juvederm or Restylane are often used. Despite their use and popularity, adverse events are known to occur from these procedures. Although most outcomes may be mild and resolve over time, rare instances of severe complications cannot be ignored, as these effects may be irreversible. Healthcare practitioners and patients must be aware of these risks, as these cosmetic procedures can affect the patient’s quality of life. The aim of this study was to evaluate the incidence of adverse events (AEs) reported from the use of hyaluronic acid dermal fillers in the facial region. A systemized search of randomized controlled trials was conducted using Cochrane Central, Embase, Medical Literature Analysis and Retrieval System Online (MEDLINE), and the Web of Science databases. After screening for eligibility and conducting a critical appraisal of the articles, 19 studies were retained for the final review. The meta-analysis results included different side effects by facial location, i.e., nasolabial fold* *(NLF) vs. other (midface, perioral line, and lip region). The midface includes the anteromedial cheek region, the zygomaticomalar region, and the submalar region. The adverse events were swelling, pain, erythema, bruising, lumps and bumps, firmness, tenderness, itching, and skin discoloration. A significant difference was found in individuals experiencing swelling, lumps or bumps, and firmness at the midface, perioral line, and lip region versus the nasolabial fold site. There was no significant difference in the proportion of individuals experiencing pain, erythema, bruising, tenderness, itching, or skin discoloration at the nasolabial fold site versus the other sites. The study highlights the prevalence of common AEs that can result from HA dermal fillers like Juvederm or Restylane, thus emphasizing the importance of healthcare professionals explaining the risk and benefits to patients.

## Introduction and background

Dermal fillers, also known as facial fillers, are soft, gel-like substances that are injected beneath the skin. They are used as a treatment choice for scars, volume deficiency, facial sculpting, rhytides, and facial augmentation and contouring [1]. Since the approval of the first dermal filler by the United States Food and Drug Administration (FDA) in 1981, there are now four groups of approved fillers: 1) hyaluronic acid (HA), 2) polymethyl methacrylate (PMMA), 3) poly-L-lactic acid, and 4) calcium hydroxylapatite (CaHA) [2-3].

Dermal fillers have proven to be extremely popular aesthetic procedures. According to the American Academy of Facial Plastic and Reconstructive Surgery, the top procedures performed on women in 2021 include neurotoxins at 63%, followed by dermal fillers at 57% [4]. The Aesthetic Society members reported that Americans spent more than three billion dollars on nonsurgical aesthetic procedures in 2020 [5]. Possible explanations for the rise in popularity of injectable procedures include short recovery times, minimally invasive techniques, effective results, and societal standards.

Types of dermal fillers

While all dermal fillers provide volume, they differ in their ingredients and mechanism of action. CaHA is a naturally occurring substance found primarily in bones. It is thicker than HA fillers, helps stimulate natural collagen production, and is recommended for treating deeper lines, smoothening chin wrinkles, and softening nasolabial folds [6]. PMMA is a synthetic compound that remains beneath the skin to provide indefinite support, contains collagen, and is recommended for softening nasolabial folds and filling acne lines [6]. Poly-L-lactic Acid is a synthetic substance, a collagen stimulator, and is recommended for smoothening nasolabial folds and treating deeper facial wrinkles [6]. HA is a natural compound found in the skin and remains the most widely used dermal filler due to its safety and effectiveness [7].

Use of hyaluronic acid dermal fillers

According to the American Board of Cosmetic Surgery, HA fillers work best to soften nasolabial folds, smooth vertical lip lines, smooth chin wrinkles, fill acne scars, plump the lips, smooth marionette lines, lift and enhance the cheek, and smooth under the eyes/tear troughs [6]. HA has limited immunogenic potential, a longer duration of effect, ease of reversibility with the use of hyaluronidase, and plays a significant role in keeping the dermis hydrated and volumized [8-11]. The types of HA fillers include Juvederm, Restylane, Voluma, and Belotero [12]. Although they all contain HA, they each have different characteristics that influence their use in clinical practice, including gel hardness, viscosity, type of crosslinker used, degree of crosslinking, total HA concentration, and lifetime in the skin [13]. Despite the rise in popularity and aesthetic benefits, the overall number of complications and adverse events (AEs) reported have also risen.

Adverse events associated with hyaluronic acid dermal fillers

Adverse events associated with HA filler treatment are mostly mild, self-limiting, and reversible. The most common complications are injection site reactions, including edema, pain, erythema, itching, and ecchymosis [14]. Other reported AEs are hypersensitivity reactions, infections, Tyndall effect, and surface irregularities and nodules [15-18]. More serious complications due to vascular occlusion have resulted in blindness and skin necrosis [14]. A recent publication suggested a guideline to help healthcare practitioners identify and manage vascular occlusions after dermal injections with the use of ultrasound [19]. The rise in complications has incited more research into the adverse events that can result from dermal fillers.

Previous studies on adverse events of dermal fillers

Various systematic reviews and meta-analyses have been conducted to investigate the effectiveness, safety, and adverse events of dermal fillers injected into the nasolabial folds [20-23]. However, each meta-analysis is distinct in that it each evaluates certain dermal fillers. One meta-analysis on the nasolabial fold incorporated a variety of dermal fillers, consisting of 51 studies with 4,097 patients [20]. The most common complications reported were lumpiness, tenderness, swelling, and bruising. Another meta-analysis investigated the effectiveness and safety of hyaluronic acid gel with lidocaine as a treatment for the nasolabial folds to diminish pain while administering the dermal filler [21]. Although there was no significant difference in the effectiveness of the HA dermal filler with and without lidocaine, the main adverse events that were reported for both groups consisted of swelling, erythema, bruising, itching, and induration. Two additional meta-analyses have been conducted evaluating the effectiveness and safety of monophasic and biphasic HA fillers for the treatment of nasolabial folds [22-23]. Both studies reported that the monophasic HA filler was more effective in the treatment of nasolabial folds. Moreover, there was no difference in the number of adverse events in both monophasic and biphasic HA fillers. Another study reported that the main AEs in both groups were swelling, erythema, bruising, itching, and induration [22]. Although these AEs are mild and resolve with time, healthcare practitioners must disclose to patients the potential complications that can result from the cosmetic procedure, as some require the use of hyaluronidase to reverse the procedure and complications.

An additional meta-analysis evaluated the effectiveness and adverse events of HA fillers on lip augmentation [24]. The adverse events reported consisted of tenderness, injection site swelling, and bruising. Rare AEs resulted in foreign body granulomas, herpes labialis, and angioedema. Although this study did not report any severe AEs, such as vascular complications, a meta-analysis utilizing case reports and case series explored the frequency and severity of vascular complications with the injection of dermal fillers [25]. The main AE reported was blindness (n=57; 61%), where only 24 cases (28%) had a partial or total recovery, and a majority of the cases (n=61; 72%) reported no improvement.

The abovementioned six studies sought to investigate the effectiveness and safety of the filler being injected. Out of those, two focused on all types of dermal fillers, while the other four evaluated only HA dermal fillers used in certain areas of the face. To our knowledge, there are no published meta-analysis reports that focus solely on the complications of HA dermal filler injections. Due to the fact that HA is the most commonly used dermal filler, studying its effects is warranted. By focusing solely on potential complications and AEs that can occur from the injection of HA dermal fillers, the current meta-analysis provides useful information to help determine the prevalence and type of AEs, highlighting potential risks. Although most outcomes may be mild and resolve over time, rare instances of severe complications cannot be ignored, as these effects may be irreversible and impede patients’ quality of life. The aim of this systematic review and meta-analysis was to evaluate the incidence of complications and AEs that result from patients receiving hyaluronic acid dermal fillers for cosmetic purposes in all areas of the facial region.

## Review

Methods

Eligibility Criteria

To meet the inclusion criteria, articles had to be primary studies that reported findings from randomized controlled trials (RCTs) or clinical trials (CTs). Of all the several ways research can be conducted, the gold standard level of proof where treatments and therapies are concerned is the RCT and the CT. These types of studies were selected to reduce bias and produce the most precise results. Additionally, the HA dermal filler procedures needed to be performed in the United States or Canada, as other countries may use dermal fillers that have not been approved by the FDA and therefore do not provide much relevance to our targeted demographic. Other inclusion criteria consisted of a study performed between the period of 2000-2022, enlisted healthy female and male participants aged 18 and older; participants received HA dermal fillers for cosmetic purposes only; participants received HA injections in the facial region; AEs were reported in detail and written in English. Articles were excluded if they were opinion pieces, retrospective studies, cohort studies, or literature reviews. Additionally, studies using fillers other than HA or studies using added materials such as Botox were excluded to ensure the AEs reported were due to the HA filler and no other substances.

Search Strategy

The first and second authors, in consultation with the university librarian, created the search strategy for online database searching. The search strategy was created using key terms from Medical Subject Headings (MeSH). The following databases were searched: Cochrane Central, Embase, MEDLINE, and Web of Science. Data collection occurred on October 11, 2022, and searches were conducted in Cochrane Central, Embase, MEDLINE, and Web of Science. The databases were selected for the following reasons: Cochrane Central for its wide range of clinical controlled trials; Embase for its up-to-date health and medical topics; MEDLINE for its extensive medical subheadings and guided MeSH subject searching; and Web of Science for finding articles that may have been missed in the other databases.

The research question was based on the population (P), intervention (I), comparison (C), and outcome (O) strategy (PICO). The P represents males and females over the age of 18, I represents patients receiving HA dermal fillers for cosmetic purposes only, C represents patients not receiving dermal fillers (no treatment), and O represents skin complications measured as yes or no. The researchers were attempting to find the incidence of reported AEs in patients receiving HA dermal fillers in the facial region.

Two reviewers searched together under the instruction of a librarian using the descriptors from MeSH. The following keyword search was used in Embase: (“dermal filler*” OR “hyaluronic acid*” OR “dermal implant*” OR “Juvederm*” OR “facial filler*”) AND (face OR chin OR nasal OR nose OR cheek OR facial OR lip). The Boolean operators AND OR were used to combine and exclude keywords in the search. The search phrase was adapted to the other three databases. “Adverse events” and “complications” were not included in the search phrase to avoid bias.

Screening and Study Selection

**Figure 1:**
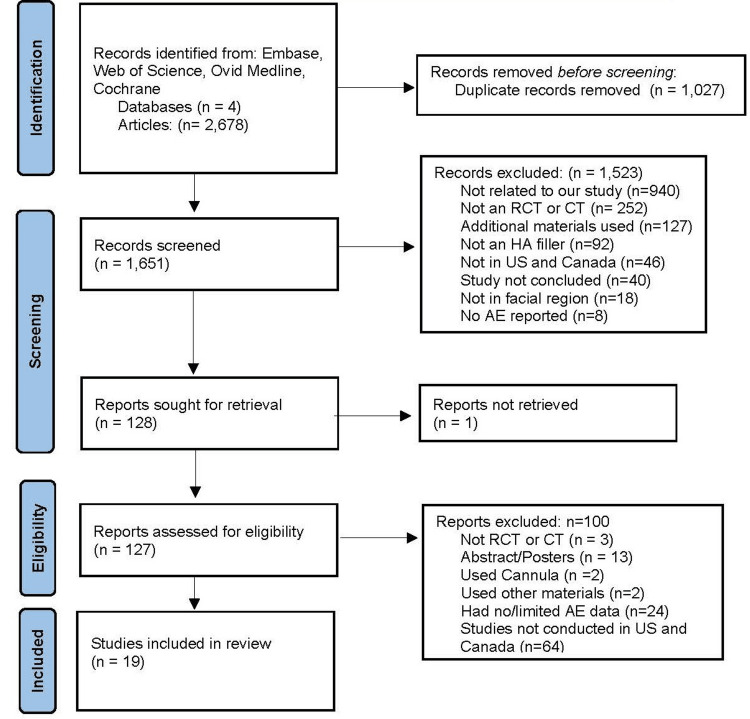
PRISMA flow diagram PRISMA: Preferred Reporting Items for Systematic Reviews and Meta-Analyses; RCT: randomized controlled trials; CT: clinical trials; HA: hyaluronic acid; AE: adverse events

The initial search yielded a total of 2,678 articles. Of those, 1,027 duplicates were removed, leaving a total of 1,651 studies to be screened. From those studies, 726 were from Embase, 698 were from MEDLINE, 817 were from Web of Science, and 437 were from Cochrane Central (437). Rayyan systematic review collaboration software (Rayyan Systems, Cambridge, Massachusetts, United States) was used to independently screen the abstracts and titles of the 1,651 articles. The two reviewers reached a consensus and classified the studies as either ‘included’ (n=128), ‘excluded’ (n=1,523), or ‘maybe’ (n=0). Articles were excluded for the following reasons: not related to the current aim of the study (n=940), not an RCT or CT (n=252), additional materials used (n=127), not an HA filler (n=92), not performed in the United States or Canada (n=46), study not concluded (n=40), not in the facial region (n=18), and no AE reported (n=8). Two researchers independently read the remaining 128 articles in detail to verify the inclusion criteria were met. If there was discordance between the two researchers on whether a particular article should be included, a third reviewer was presented the article and involved in the discussion until an agreement was reached. This process yielded 100 articles to be excluded for the following reasons: not an RCT or CT (n=3), abstract/posters (n=13), used cannula (n=2), used other materials (n=2), had no or limited AE data (n=24), and not conducted in the United States or Canada (n=64). As a result, 19 studies were chosen for the study. The Preferred Reporting Items for Systematic Reviews and Meta-Analysis (PRISMA) method in Figure 1 details the screening and selection process.

Selection of Sources of Evidence

To increase consistency among reviewers, both reviewers one and two independently screened the same 1,651 articles for abstracts and titles and read the full text of the remaining 128 articles to assess if the inclusion criteria were met. Disagreements were resolved during the study selection and data extraction process by sharing rationales for inclusion/exclusion and involving a third reviewer if needed until an agreement was reached. Consequently, 19 articles were identified to be most relevant and were thus used for the final analysis.

Data Charting Process

A data extraction charting form was developed by the team using Microsoft Excel (Microsoft Corporation, Redmond, Washington, United States) to extract relevant information from the 19 included articles. Two reviewers independently charted the data, discussed the results, and continually updated the data-charting form. If there was discordance between the two researchers on whether a particular variable should be included, a third reviewer was presented with the article and involved in the discussion until an agreement was reached. The following sections were included in the extraction form: title of the article, author and year, number of participants, location on the face, and the reported outcomes. Although other AEs were present in the studies, outcomes were limited to nine (the most frequently reported in the 19 studies). Each AE (swelling, pain, erythema, bruising, lumps/bumps, firmness, tenderness, itching, skin discoloration) was categorically described as present or absent and numerically recorded. The data extraction information was then sent to another member of the research team for further statistical analysis.

Quality Appraisal

The Joanna Briggs Institute Appraisal Tools (JBI) were used to perform a methodological quality assessment and reduce the risk of bias in the included studies. All 19 articles selected for the meta-analysis were extensively analyzed by two researchers for reliability and relevance using the JBI critical appraisal checklist for RCTs and CTs. The appraisal checklist helped to ensure the final articles contained a control and treatment group, that participants were randomized, that evaluators and patients were blinded, and appropriate RCT protocols were followed. The researchers answered the questions on the checklist with yes, no, or unclear and then selected whether to include, exclude, or seek further information for each study. After review, the critical appraisers met on an online conference call to compare their results. The articles were classified as high, moderate, or low risk of bias: score less than 50%, 50-70%, and score above 70% respectively. All articles had a low risk of bias and were approved for inclusion in the study.

Data Synthesis

An overall proportion of studies reporting different side effects by facial location, i.e., nasolabial fold (NLF) vs. other (midface, perioral line, and lip region), was calculated. The midface includes the anteromedial cheek, zygomaticomalar, and submalar regions. The side effects were swelling, pain, erythema, bruising, lumps and bumps, firmness, tenderness, itching, and skin discoloration. Fourteen different fillers were used including Juvederm Ultra XC, Belotero Balance, Juvederm Ultra Plus, Juvederm Volbella XC, Juvederm Vollure XC, Juvederm Voluma XC, Perlane, Perlane-L, Resilient Hyaluronic Acid Redensity, Resilient Hylaruonic Acid (RHA), Restylane, Restylane Defyne, Restylane Lyft, and Restylane-L. We used a generalized linear mixed model (GLMM) for pooling because of the variability in side effects and location. Moreover, a GLMM model allows for the generalization of the results at the population level. To evaluate the studies for heterogeneity, chi-square tests and I2statistic were utilized. A value of 0.05 was considered statistically significant for the chi-square tests and I2 values ≥ 75 were indicative of high heterogeneity. Nineteen studies were used in the analysis.

Results

Table 1 reports the characteristics of the 19 studies included in the review. 

**Table 1:** Characteristics of the included studies *Only outcomes present were listed in the outcomes column **Studies combined the adverse events for both fillers HA: hyaluronic acid; JUP: Juvederm Ultra Plus; RD: Restylane Defyne; P: Perlane; P-L: Perlane-L; BB: Belotero Balance; JU XC: Juvederm Ultra XC; NASHA: Non-Animal Stabilized Hyaluronic Acid; R: Restylane; JV XC: Juvederm Volbella XC;  R-L: Restylane-L; NLF: nasolabial folds; RHA: Resilient HA; RHAR: Resilient Hyaluronic Acid Redensity; SGP-HA: small gel particle hyaluronic acid; HYC-24L: Juvéderm Ultra XC, a 24 mg/mL hyaluronic acid gel containing 0.3% (wt/wt) lidocaine; CPM-22.5: Belotero Balance, 22.5 mg/mL of hyaluronic acid

Author	Study Design	Study Aim	Filler and units injected during initial treatment	Location on the face	Number of Treatment participants	Patient Characteristics	Outcomes*
Baumann, 2018 [26]	Parallel, randomized, subject and evaluator-blinded, active- controlled, intraindividual split-face comparison study	To compare the efficacy and safety of HA gel with lidocaine and HA gel without lidocaine in the treatment of moderate to severe nasolabial folds.	Juvederm Ultra Plus (JUP): 1.45mL Restylane Defyne (RD): 1.39 mL	Nasolabial Fold	n=136 (JUP) n=136 (RD)	M= 53.7 Range: 34-75	Juvederm Ultra Plus: Swelling: n=3; Pain: n=2; Erythema: n=3 Restylane Defyne: Swelling: n=4; Pain: n=4; Erythema: n=3
Beer, 2015 [27]	Randomized, no-treatment controlled, evaluator-blinded	To compare the safety and effectiveness of small particle hyaluronic acid plus lidocaine versus no treatment for lip augmentation and perioral rhytides.	Restylane: 2.179 mL	Lip and perioral	n=218	M= 45.5	Swelling: n=94 Pain: n=21
Brandt, 2010 [28]	Randomized, double-blind, split-face study	To compare the pain relief and safety of large gel particle hyaluronic acid plus 0.3% lidocaine with that of large gel particle hyaluronic acid without lidocaine during correction of nasolabial folds and to assess filler safety in different skin types.	Perlane (P): 1.10 mL Perlane-L (P-L): 1.11 mL	Nasolabial Fold	n=60 (P) n= 60 (P-L)	M= 53.4 SD: ± 8.0	Perlane: Swelling: n=24; Pain: n=44; Erythema: n=25; Bruising: n=23; Lumps/bumps: n=1; Itching: n=5; Skin discoloration: n=0 Perlane-L: Swelling: n=24; Pain: n=44; Erythema: n=24; Bruising: n=19; Lumps/bumps: n=1; Itching: n=9; Skin discoloration: n=1
Butterwick, 2015 [29]	Randomized, controlled, Subject and evaluator blinded	Effectiveness and safety of HYC-24L and CPM-22.5 for the treatment of perioral lines	Belotero Balance (BB): 1.32 mL Juvederm Ultra XC (JU XC): 1.18 mL	Perioral	n= 136 (BB) n=136 (JU XC)	M= 58.2 SD: ± 8.4	Belotero Balance: Swelling: n=58; Pain: n=36; Erythema: n=43; Bruising: n=62; Lumps/bumps: n=46; Firmness: n=48; Tenderness: n=45; Itching: n=9; Skin discoloration: n=18 Juvederm Ultra XC: Swelling: n=59; Pain: n=28; Erythema: n=43; Bruising: n=59; Lumps/Bumps: n=52; Firmness: n=49; Tenderness: n=49; Itching: n=15; Skin discoloration: n=12
Dayan, 2015 [30]	Single-blind, randomized, no-treatment controlled study	To assess the safety and effectiveness of Juvederm Ultra XC, a 24 mg/mL hyaluronic acid gel containing 0.3% lidocaine (HYC-24L) for augmentation of the lips.	Juvederm Ultra XC: 2.13 mL	Lip and perioral	n=157	M= 49 Range: 20-79	Swelling: n=150; Bruising: n=147; Lumps/bumps: n=48; Firmness: n=141
Dayan, 2020 [31]	Prospective, randomized, within-patient controlled study, evaluator- blinded	To evaluate the safety and effectiveness of Vollure for correction of moderate to severe nasolabial folds over 18 months and after repeat treatment.	Juvederm Vollure XC: 1.7 mL	Nasolabial Fold	n=123	M= 54.0 Range: 33-83	Swelling: n=105; Pain: n=88; Erythema: n=90; Bruising: n=69; Lumps/bumps: n=100; Firmness: n= 108; Tenderness: n=103; Itching: n=38; Skin discoloration: n=33
Dover, 2009 [32]	Blinded, prospective, randomized subject and evaluator	To report the efficacy, durability, and safety data of a large particle NASHA filler and a small particle NASHA filler.	Perlane (P): 2.3 mL Restylane (R): 2.4 mL	Nasolabial Fold	n=141 (P) n=142 (R)	53.7 ± 9.0 54.4 ± 8.1	Perlane: Swelling: n=9; Pain: n=1; Erythema: n=4; Bruising: n=36; Lumps/bumps: n=3; Tenderness: n=5; Itching: n=0; Skin discoloration: n=0 Restylane: Swelling: n=5; Pain: n=1; Erythema: n=2; Bruising: n=41; Lumps/bumps: n=3; Tenderness: n=7; Itching: n=1; Skin discoloration: n=1
Few, 2015 [33]	Single-blind, randomized controlled study	The effectiveness of Juvederm Voluma XC was examined from the patient perspective.	Juvederm Voluma XC: 5.09 mL	Midface	n=235	M= 56 Range: 35-65	Swelling: n=200; Pain: n=155; Erythema: n=154; Bruising: n=181; Lumps/bumps: n=189; Firmness: n=192; Tenderness: n=215; Itching: n=90; Skin discoloration: n=96
Geronemus, 2017 [34]	Prospective, randomized, controlled, evaluator blinded	To evaluate the safety and effectiveness of VYC-15L for lip and perioral enhancement versus a nonaminal stabiliaed HA with lidocaine.	Juvederm Volbella XC (JV XC): 2.6 mL Restylane-L (R-L): 2.6 mL	Lips	n=168 (JV- XC) n=56 (R-L)	Juvederm Volbella XC: M: 53 Range: 22-78 Restylane-L: M= 55 Range: 23-75	Juvederm Volbella XC: Swelling: n=159; Pain: n=19; Bruising: n=30; Lumps/bumps: n=153; Firmness: n=153 Restylane-L: Swelling: n=54; Pain: n=11; Bruising: n=10; Lumps/bumps: n= 50; Firmness: n= 52
Glogau, 2012 [35]	Randomized, no treatment controlled, evaluator blinded study	To assess the effectiveness and safety of small gel particle hyaluronic acid for lip augmentation.	Restylane: 1.2 mL	Lips	n=135	47.8 ± 10.5	Swelling: n=42; Pain: n=15; Erythema: n=3; Lumps/bumps: n=5; Tenderness: n=4;
Jones, 2013 [36]	Evaluator-blind, randomized controlled study	To study the safety and effectiveness of a new 20 mg/mL HA gel specifically formulated and optimized for mid-face volumizing.	Juvederm Voluma XC: 5.07 mL	Midface	n=235	M= 55.0 Range: 35-65	Swelling: n=201; Firmness: n=193; Lumps/bumps: n=191; Tenderness: n=216
Kaufman, 2019 [37]	Prospective, multicenter, controlled, randomized, double-blind, within subject (split-face) clinical trial	The efficacy and safety of one of these resilient HA fillers, and its noninferiority to an effective comparator available in the US, were testing in the treatment of dynamic wrinkles.	Restylane Lyft: 1.42 mL RHA4: 1.54 mL	Nasolabial Fold	n=120	57.4 ± 10.0	** Swelling: n=27; Firmness: n=49; Lumps/bumps: n=55; Tenderness: n=21
Monheit, 2018 [38]	Prospective, multicenter, randomized, within-subject controlled study, double-blind	To evaluate the safety and effectiveness of VYC-17.5L for correction of moderate to severe nasolabial folds (NLF) compared with a control HA dermal filler.	Juvederm Vollure XC: 1.4 mL	Nasolabial Fold	n=122	M= 54 Range: 33-83	Swelling: n=105; Pain: n=88; Erythema: n=90; Bruising: n= 69; Lumps/bumps: n=100; Firmness: n=108; Tenderness: n=103; Itching: n=38; Skin discoloration: n=33
Monheit.2020 [39]	Prospective, multicenter, active- controlled, randomized, double-blinded, within-subject (split-face) trial	The efficacy, durability, and safety of 2 of these RHA fillers and their noninferiority to an effective HA comparator available in the US, were tested in the treatment of dynamic facial wrinkles.	RHA2/Juvderm: 1.54 mL RHA3/Juvederm: 1.52 mL	Nasolabial Fold	n=148	55.4 ± 11.0	** Swelling: n=16; Lumps/bumps: n=35; Firmness: n=40; Tenderness: n=18
Rivkin, 2019 [40]	Prospective, multicenter, controlled study, evaluator-blind	To evaluate safety and effectiveness of repeat treatment with VYC-15L administered 1 year after treatment for lip and perioral enhancement.	Juvederm Volbella XC: 0.95 mL	Lips and perioral	n=120	M= 53 Range: 22-78	Swelling: n=112; Pain: n=92; Erythema: n=90; Bruising: n=98; Lumps/bumps: n=102; Firmness: n=102; Tenderness: n=108; Itching: n=33; Skin discoloration: n=41
Sundaram, 2022 [41]	Randomized, evaluator- blind, no treatment control, multicenter, prospective clinical trial	To demonstrate superiority of RHA_R_ over no-treatment control for correction of moderate to severe dynamic perioral rhytides.	Resilient Hyaluronic Acid Redensity: 2.0 mL	Lips and perioral	n=199	61.6 ± 7.2	Swelling: n=146; Pain: n=54; Erythema: n=131; Bruising: n=154; Lumps/bumps: n=115; Firmness: n=115; Tenderness: n=105; Itching: n=31; Skin discoloration: n=94
Taylor, 2009 [42]	Prospective, randomized, split-face, patient blinded, and evaluator blinded, comparative, multicenter study	To compare the safety and efficacy of two variable particle NASHA fillers in the correction of nasolabial folds in patients with Fitzpatrick skin types IV, V and VI.	Restylane (R): 3mL Perlane (P): 3mL	Nasolabial Fold	n=150 (R) n=150 (P)	Range: 18 - 75	Restylane: Swelling: n=4; Pain: n=3; Erythema: n=16; Bruising: n=10; Lump/bumps: n=1; Tenderness: n=4; Itching: n=3; Skin discoloration: n=10 Perlane: Swelling: n=4; Pain: n=3; Erythema: n=16; Bruising: n=12; Lump/bumps: n=0; Tenderness: n=4; Itching: n=1; Skin discoloration: n=8
Weiss, 2010 [43]	Randomized, double-blind, split face study	To compare the pain relief and safety of small gel particle HA plus 0.3% lidocaine hydrochloride with that of SGP-HA without lidocaine during correction of nasolabial folds and to assess filler safety in different skin types.	Restylane (R): 1.23 mL Restylane-L (R-L): 1.24 mL	Nasolabial Fold	n=60 (R) n=60 (R-L)	52.1 ± 6.6	Restylane: Swelling: n=22; Pain: n=44; Erythema: n=27; Bruising: n=19; Lumps/bumps: n=2; Itching: n=4 Restylane-L: Swelling: n=24; Pain: n=40; Erythema: n=28; Bruising: n= 23; Lumps/bumps: n=1; Itching: n=6
Weiss, 2016 [44]	Evaluator-blind, randomized trial	To evaluate whether large gel particle hyaluronic acid with lidocaine is more effective in the treatment of midface deficiencies than no treatment.	Restylane: 6.23 mL	Midface	n=199	M= 52.6	Swelling: n=15; Pain: n=17; Bruising: n=36

Swelling

**Figure 2:**
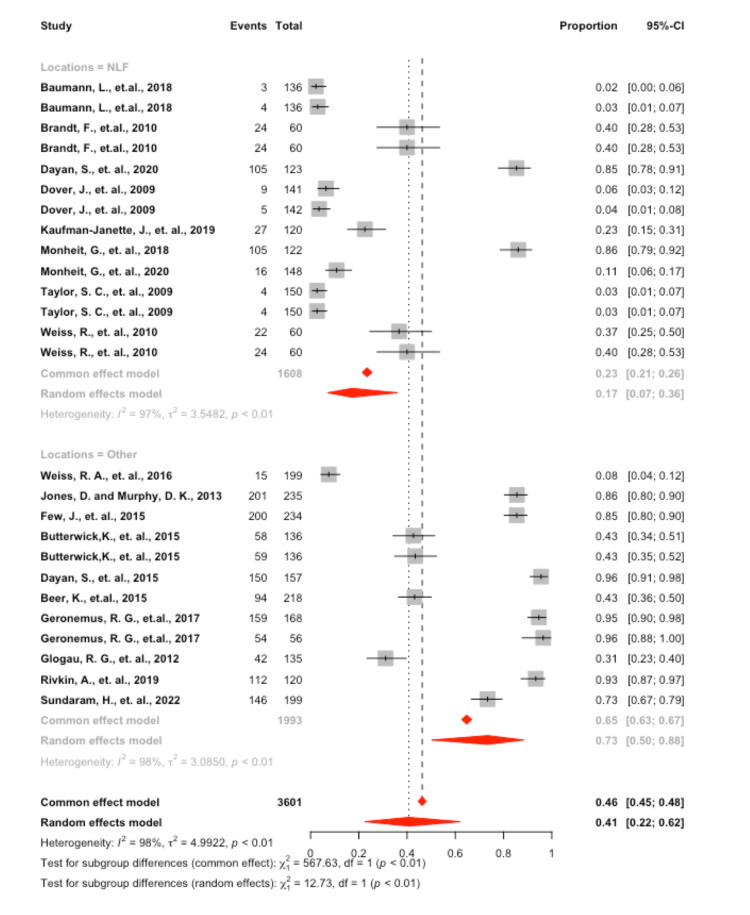
Forrest plot of swelling by location

A significant amount of heterogeneity was found in the model, I2 = 98.0% (p<0.001), which justified employing the random effects model. Interpreting the mixed effects model, it was found that the overall proportion of swelling was 40.7 (95 CI: 22.3; 62.1). A significant difference was found in the proportion of individuals experiencing swelling found at the NLF site 17.1 (95 CI: 7.0; 36.1) vs. other sites 73.4 (95 CI: 50.1; 88.3), p = 0.004 (Figure 2).

Pain

A significant amount of heterogeneity was found in the model, I2 = 97.6% (p<0.001), which justified employing the random effects model. Interpreting the mixed effects model, it was found that the overall proportion of individuals experiencing pain was 9.5 (95 CI: 3.3; 24.1). No significant difference was found for individuals experiencing pain at the NLF site 7.6 (95 CI: 1.4; 32.1) vs. other sites 12.3 (95 CI: 3.9; 32.4), p = 0.627.

Erythema

A significant amount of heterogeneity was found in the model, I2 = 95.0% (p<0.001), which justified employing the random effects model. Interpreting the mixed effects model, it was found that the overall proportion of individuals experiencing erythema was 4.5 (95 CI: 1.1; 15.9). No significant difference was found in the proportion of individuals experiencing erythema at the NLF site 9.9 (95 CI: 2.9; 28.8) vs. other sites 0.02 (95 CI: 0.01; 19.4), p = 0.167.

Bruising

A significant amount of heterogeneity was found in the model, I2 = 95.5% (p<0.001), which justified employing the random effects model. Interpreting the mixed effects model, it was found that the overall proportion of individuals experiencing bruising was 10.8 (95 CI: 3.2; 30.6). No significant difference was found in the proportion of individuals experiencing bruising at the NLF site 7.9 (95 CI: 1.8; 28.0) vs. other sites 16.3 (95 CI: 2.3; 61.0), p = 0.531.

Lumps Bumps

**Figure 3:**
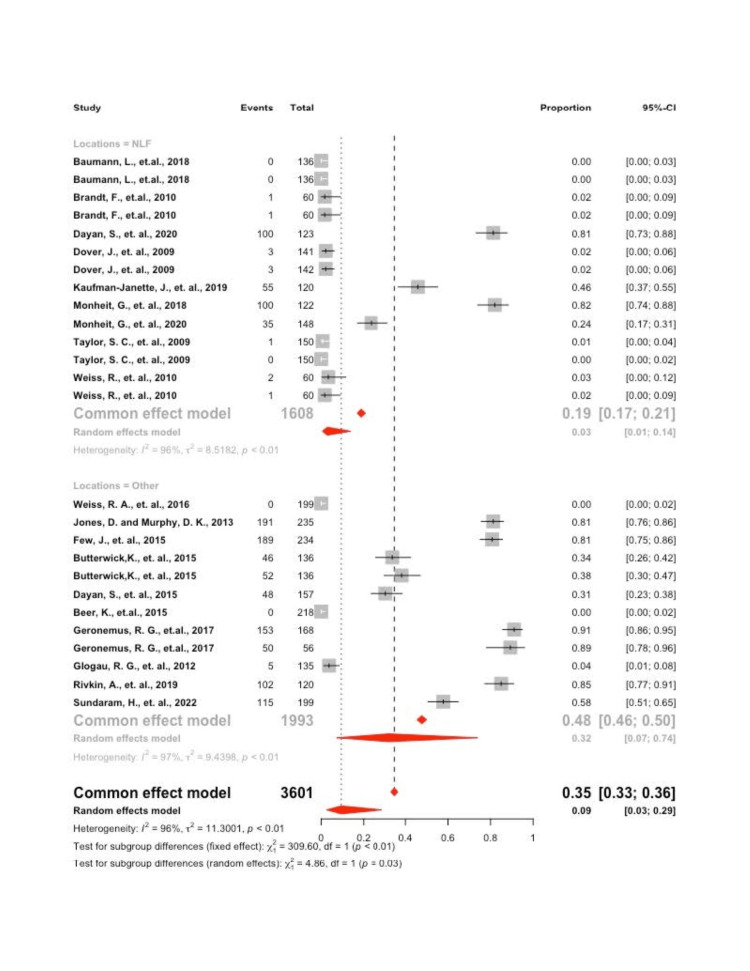
Forrest plot of lumps and bumps by location

A significant amount of heterogeneity was found in the model, I2 = 96.3% (p<0.001), which justified employing the random effects model. Interpreting the mixed effects model, it was found that the overall proportion of individuals experiencing lumps and bumps was 9.4 (95 CI: 2.6; 28.8). A significant difference was found in the proportion of individuals experiencing lumps and bumps at the NLF site 2.9 (95 CI: 0.01; 13.8) vs. other sites 32.1 (95 CI: 7.3; 73.9), p = 0.027 (Figure 3).

Firmness

**Figure 4:**
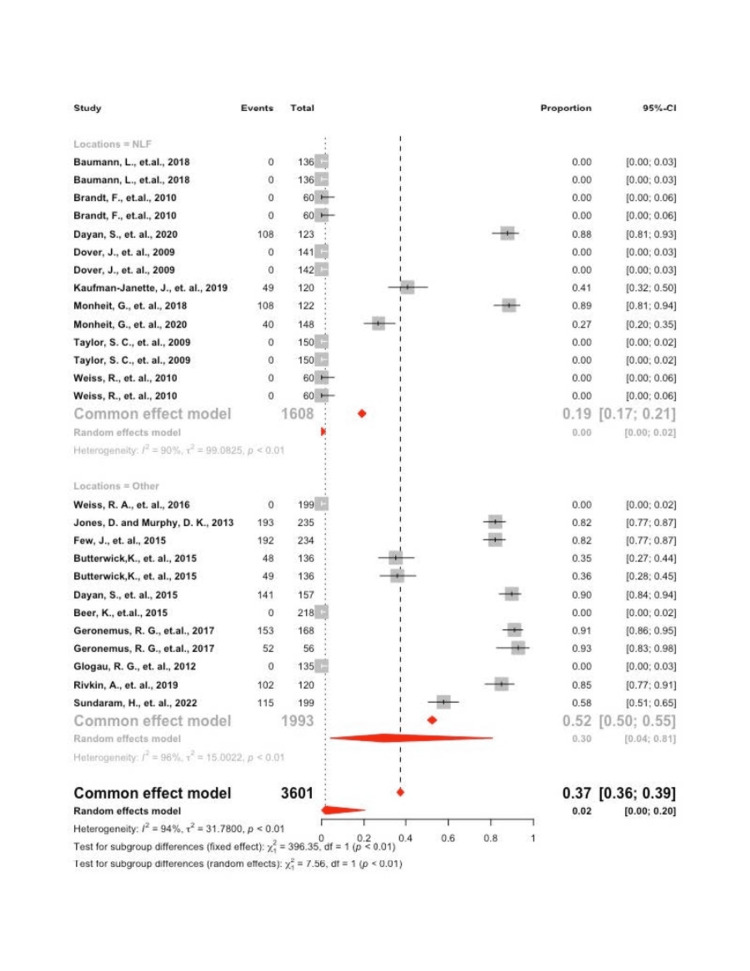
Forrest plot of firmness by location

A significant amount of heterogeneity was found in the model, I2 = 96.3% (p<0.001), which justified employing the random effects model. Interpreting the mixed effects model, it was found that the overall proportion of individuals experiencing firmness was 1.7 (95 CI: 0.1; 20.3). A significant difference was found in the proportion of individuals experiencing firmness at the NLF site 0.01 (95 CI: 0.00; 0.02) vs. other sites 29.6 (95 CI: 0.04; 80.7), p = 0.006 (Figure 4).

Tenderness

A significant amount of heterogeneity was found in the model, I2 = 96.6% (p<0.001), which justified employing the random effects model. Interpreting the mixed effects model, it was found that the overall proportion of individuals experiencing tenderness was 2.2 (95 CI: 0.1; 12.9). No significant difference was found in the proportion of individuals experiencing tenderness at the NLF site 1.7 (95 CI: 0.02; 12.2) vs. other sites 3.3 (95 CI: 0.01; 45.9), p = 0.730.

Itching

A significant amount of heterogeneity was found in the model, I2 = 82.8% (p<0.001), which justified employing the random effects model. Interpreting the mixed effects model, it was found that the overall proportion of individuals experiencing itching was 0.8 (95 CI: 0.1; 3.5). No significant difference was found in the proportion of individuals experiencing itching at the NLF site 1.4 (95 CI: 0.03; 6.2) vs. other sites 0.03 (95 CI: 0.01; 6.3), p = 0.394.

Skin Discoloration

A significant amount of heterogeneity was found in the model, I2 = 85.4% (p<0.001), which justified employing the random effects model. Interpreting the mixed effects model, it was found that the overall proportion of individuals experiencing skin discoloration was 0.03 (95 CI: 0.01; 2.3). No significant difference was found in the proportion of individuals experiencing skin discoloration at the NLF site 0.03 (95 CI: 0.01; 3.3) vs. other sites 0.03 (95 CI: 0.01; 8.6), p = 0.965.

Discussion 

The presented study aimed to evaluate the common adverse events reported from hyaluronic acid dermal filler injections to the facial region. The results yielded both significant and non-significant findings. Foremost, a significant difference was found in the proportion of individuals experiencing swelling, lumps or bumps, and firmness at the midface, perioral line, and lip region versus the nasolabial fold site. The midface includes the anteromedial cheek, zygomaticomalar, and submalar regions. Additionally, there was no significant difference in the proportion of individuals experiencing pain, erythema, bruising, tenderness, itching, or skin discoloration at the nasolabial fold site versus the other sites.

Although dermal fillers are considered relatively safe, AEs can occur. The results did not yield any unexpected findings; other meta-analysis studies reported similar AEs to those found in the current study. Stefura et al. focused on the effectiveness and safety of several types of dermal fillers localized in the nasolabial fold area [20]. Their results were similar to the current study, as the most common AEs were mild and reversible and included lumpiness (43%), tenderness (41%), swelling (34%), bruising (29%), pain (28%), and redness (26%). Another meta-analysis assessed the effectiveness of hyaluronic acid as a dermal filler for lip augmentation [24]. Their analysis revealed that the most frequent AEs were injection-related, such as tenderness, swelling, bruising, mass, and pain. Another meta-analysis evaluated the difference between monophasic vs. biphasic hyaluronic acid fillers for correcting nasolabial folds [23]. The study found injection site pain to be the main complaint after receiving dermal fillers. Additionally, there were no differences reported in adverse events between monophasic hyaluronic acid (MHA) and biphasic hyaluronic acid (BHA) fillers. Wang et al. studied the effect of incorporating lidocaine with HA to provide a pain-relieving alternative [21]. The study showed that the use of lidocaine was more effective for pain relief than HA alone. However, both resulted in similar effectiveness and safety for treating the NLF. The common complications reported in the study were swelling, erythema, bruising, itching, and induration [21]. Lastly, Peng et al. found no significant difference in the incidence of adverse events between monophasic and biphasic hyaluronic acid fillers in the NLF region [22]. These findings suggest complications resulting from dermal filler injections may often be directly related to the injection site technique rather than the type of HA filler [45].

Although the AEs reported in the current analysis met the researchers’ expectations, the results posed an interesting question as to why the nasolabial fold region experiences less swelling, lumps or bumps, and firmness than the midface area, perioral lines, or lip region. One possible explanation could be the injection site technique used in the studies. The nasolabial fold is a particularly sensitive area, as the facial artery runs along its course. It is important to inject the filler medial to the nasolabial fold in the subdermal plane. Lumps or bumps typically arise due to incorrect superficial placement of the filler [46]. An experienced medical professional should know how to massage the area after placement. Swelling is a common complication after filler injection, as hyaluronic acid attracts and binds water to the tissues [11]. Swelling, redness, and bruising are normal physiological responses to a foreign substance being introduced to the body [47]. A clinician can mitigate these effects by advising the patient to gently apply an ice pack to cool the injected area. The swelling should dissipate a few days after treatment, but if it persists, the patient must contact the healthcare professional, as this is not a normal reaction.

Another possible explanation for why the nasolabial fold region experiences less AEs than the midface area, perioral lines, or lip region could be that the anatomy of the nasolabial fold is less susceptible to these reactions. Similar to these results, Rayess et al. found the most common location for adverse events arose from the cheek (43%) or the lip (30%) [48]. Their study also reported more serious adverse reactions, including blindness and dermal necrosis [48]. Other possible explanations for more swelling, lumps or bumps, and tenderness to the midface, perioral line, and lip region could stem from the needle size, filler volume, type of HA filler, needle versus cannula, and experience by the clinician.

The present study highlights the significance of paying attention to the anatomy and correct technique when applying dermal fillers to the facial region, especially the midface area, perioral lines, and lip region. Although no serious adverse events were reported in the current study, Alam et al. investigated the risk of vascular occlusion with needles vs. cannulas and found the nasolabial fold to be one of the anatomic sites at which occlusions were commonly reported, along with the glabella [49]. Additionally, other researchers found autologous fat and HA fillers were among the most frequently involved in vascular occlusions [25]. The advantage of HA is the availability of hyaluronidase, an effective reversible procedure that can be used in case of an emergency [50]. The presented study highlights the importance of healthcare professionals having proficient knowledge of anatomy and dermatological practices.

From this study, it is suggested that the common AEs that result from HA fillers are usually mild, reversible, and expected to occur. Healthcare professionals usually explain to their patients that, after injecting dermal fillers, they may experience swelling, pain, erythema, bruising, lumps or bumps, firmness, tenderness, itching, and skin discoloration [51]. Also, the midface area, perioral lines, and lip region seem to be more prone to AEs than other areas of the face, such as the NLF. However, this finding may be due to the current study containing more reports involving the NLF region than other sections of the face due to the majority of the published data that satisfied the inclusion criteria focused on this region.

Limitations

This study has several limitations, and its results must be considered with caution. First, only RCTs and CTs were included. These studies were chosen for inclusion due to the restrictions and proper protocols that are followed when conducting a meta-analysis. However, most of the studies that report serious adverse events such as vascular issues, cutaneous necrosis, or blindness are case studies or observational studies, which could not be included [14,25,52-53]. This perhaps minimized the relevance of the current results, as more serious complications reported in those studies had to be disregarded. Second, only RCTS and CTS conducted in the United States and Canada were analyzed. Therefore, studies that took place in other parts of the world that were excluded may have reported more severe AEs. Third, this study did not evaluate AEs between different HA fillers, injection site techniques, needle size, filler volume, utilization of a needle vs. a cannula, and clinician experience. These abovementioned factors may have played a role in AEs that could have resulted from HA fillers. Lastly, hardly any RCTs or CTs that focused on the chin or glabella region could not be found that met our inclusion criteria; these facial areas were thus not included due to limited data. The RCTs that did include the chin or glabella region did not report the same adverse events as our included studies, thus would not allow us to run an effective analysis [54-55].

Recommendations for future practice

It is recommended that healthcare professionals take the time to educate patients thoroughly on the possible AEs that may result from HA fillers. Although a protocol exists for healthcare professionals to follow, the focus tends to be on explaining the possible overall AEs and how they might impact the patient's quality of life. However, it might be advantageous to deliver targeted explanations regarding the benefits and risks of injecting dermal fillers in various areas of the face, as each area may have a different likelihood of resulting in a reaction. Moreover, prior to commencing the procedure, healthcare professionals should ensure that the patients fully understand and are aware of the risks of this elective and cosmetic procedure [56]. Lastly, healthcare professionals should stay up to date on the latest dermal filler techniques and strategies that have been proven to minimize adverse events from HA fillers, as included in this study: proper placement of the filler [51], using an infrared device, proper instrument, and proper injection technique [57].

Recommendations for future research

Recent literature has proposed the use of ultrasound while injecting dermal fillers to ensure the safest placement [58-60]. The ultrasound allows the healthcare professional to visualize the location prior to injecting the filler and can help identify any surrounding anatomy that may be affected. Each patient has a heterogeneous anatomy of the face, which means an injection technique that may work for one patient may not work for the next [59-60]. More RCTs and CTs are needed to investigate whether the use of ultrasound can lessen the likelihood of severe adverse events related to dermal fillers. For instance, Rocha et al. proposed a three-step HA filling technique to ensure greater safety against vascular occlusion using ultrasound: prior arterial mapping, real-time U.S.-guided filling, and assessing perfusion [59]. Future research should also investigate adverse events between different HA fillers, injection site techniques, needle size, filler volume, utilization of a needle vs. a cannula, and experience by the clinician. Any of these factors can contribute to AEs resulting from dermal fillers.

## Conclusions

Dermal fillers are known for their ability to enhance one's appearance and increase self-esteem, yet one rarely hears of the repercussions that result from dermal fillers. The goal of this systematic review and meta-analysis was to report the common AEs that may result from dermal fillers in different areas of the face. A significant difference was found in individuals experiencing swelling, lumps or bumps, and firmness at the midface, perioral line, and lip region versus the nasolabial fold site. However, there was no significant difference in pain, erythema, bruising, tenderness, itching, or skin discoloration at the nasolabial fold site versus the other sites. Future clinical research should focus on investigating the prevalence of severe AEs resulting from dermal fillers, factors that provoke AEs, and strategies to reduce AEs with devices such as ultrasound and more rigorous aesthetic training. More RCTs and CTs are needed to investigate vascular complications from dermal filler injections. Although vascular complications are rare, healthcare professionals must be aware of this serious complication and how to treat it. Dermal fillers are meant to enhance one’s physical appearance and self-confidence; thus, it is the responsibility of the healthcare professional to ensure that all AEs are minimal while providing the enhancement the patient is looking for.
