# Correction: Adverse Events Reported From Hyaluronic Acid Dermal Filler Injections to the Facial Region: A Systematic Review and Meta-Analysis

**DOI:** 10.7759/cureus.c125

**Published:** 2023-06-30

**Authors:** Jessica Colon, Sophia Mirkin, Patrick Hardigan, Matthew J Elias, Robin J Jacobs

**Affiliations:** 1 Dr. Kiran C. Patel College of Osteopathic Medicine, Nova Southeastern University, Fort Lauderdale, USA; 2 Health Professions Division, Nova Southeastern University, Dr. Kiran C. Patel College of Allopathic Medicine, Fort Lauderdale, Fl, USA; 3 Dermatology, Elias Dermatology, LLC, Fort Lauderdale, USA

This article has been corrected at the request of the authors due to several proofreading errors that occurred during statistical analysis interpretation. The errors do not invalidate the work and have no impact on the statistical analysis, results, or conclusions of the study. However, in the interest of accuracy and clarity, the authors have requested that these corrections be made and the journal agrees.

The proofreading errors were corrected throughout the article in reference to the finding regarding significant differences between individuals experiencing swelling, lumps or bumps, and firmness at the midface, perioral line, lip region and the nasolabial fold site. The corrections are listed below:

In the abstract: *A significant difference was found in individuals experiencing swelling, lumps or bumps, and firmness at the nasolabial fold site versus the midface, perioral line, and lip region*.” should be “A significant difference was found in individuals experiencing swelling, lumps or bumps, and firmness at the midface, perioral line, and lip region versus the nasolabial fold site.” In the discussion: *“Foremost, a significant difference was found in the proportion of individuals experiencing swelling, lumps or bumps, and firmness at the nasolabial fold site versus the midface perioral line, and lip region”* should be “Foremost, a significant difference was found in the proportion of individuals experiencing swelling, lumps or bumps, and firmness at the midface, perioral line, and lip region versus the nasolabial fold site.”   

*"Although the AEs reported in the current analysis met the researchers’ expectations, the results posed an interesting question as to why the nasolabial fold region experiences more swelling, lumps or bumps, and firmness than the midface area, perioral lines, or lip region*." should be "Although the AEs reported in the current analysis met the researchers’ expectations, the results posed an interesting question as to why the nasolabial fold region experiences less swelling, lumps or bumps, and firmness than the midface area, perioral lines, or lip region." *“Another possible explanation for why the nasolabial fold region experiences more AEs than the midface area, perioral lines, or lip region could be that the anatomy of the nasolabial fold is more susceptible to these reactions. However, contrary to the current results, Rayess et al. found the most common location for adverse events arose from the cheek (43%) or the lip (30%) [48*]” should be “Another possible explanation for why the nasolabial fold region experiences less AEs than the midface area, perioral lines, or lip region could be that the anatomy of the nasolabial fold is less susceptible to these reactions. Similar to these results, Rayess et al. found the most common location for adverse events arose from the cheek (43%) or the lip (30%) [48].” *“Other possible explanations for more swelling, lumps or bumps, and tenderness to the NLF region could stem from the needle size, filler volume, type of HA filler, needle versus cannula, and experience by the clinician.”* should be “Other possible explanations for more swelling, lumps or bumps, and tenderness to the midface, perioral line, and lip region could stem from the needle size, filler volume, type of HA filler, needle versus cannula, and experience by the clinician.” *“The present study highlights the significance of paying attention to the anatomy and correct technique when applying dermal fillers to the facial region, especially the NFL region”* should be “The present study highlights the significance of paying attention to the anatomy and correct technique when applying dermal fillers to the facial region, especially the midface area, perioral lines, and lip region.” *“Also, the NLF region seems to be more prone to AEs than other areas of the face, such as the midface area, perioral lines, and lip region”* should be “Also, the midface area, perioral lines, and lip region seem to be more prone to AEs than other areas of the face, such as the NLF.”

In the conclusion: *“A significant difference was found in individuals experiencing swelling, lumps or bumps, and firmness at the nasolabial fold site versus the midface, perioral line, and lip region”* should be “A significant difference was found in individuals experiencing swelling, lumps or bumps, and firmness at the midface, perioral line, and lip region versus the nasolabial fold site.” 

